# Epstein–Barr Virus-Positive Langerhans Cell Sarcoma: Is There a Link? A Case Report

**DOI:** 10.3389/fonc.2021.769310

**Published:** 2022-01-18

**Authors:** Yu Guo, Shui-Hong Zhou, Zai-Zai Cao, Yang-Yang Bao, Li-Fang Shen, Hong-Tian Yao

**Affiliations:** ^1^ Department of Otolaryngology, The First Affiliated Hospital, College of Medicine, Zhejiang University, Hangzhou, China; ^2^ Department of Pathology, The First Affiliated Hospital, College of Medicine, Zhejiang University, Hangzhou, China

**Keywords:** Langerhans cell sarcoma, EBV, immunosuppression, cervical giant cyst, CD56

## Abstract

Langerhans cell sarcoma (LCS) is an extremely rare, malignant neoplasm that originates from Langerhans cells (LCs). Fewer than 70 cases have been reported in the English-language literature. LCS typically involves multiple organs, including the skin, lymph nodes, lungs, bone, bone marrow, liver, spleen, and soft tissues. Several etiological factors for LCS have been proposed, including immunosuppression, virus infection, and prior hematological disease. We report a rare case of LCS with Epstein–Barr virus (EBV) infection; bilateral cervical giant cysts were the initial manifestation. To our knowledge, this is the first report of LCS with EBV infection. The case information was complete, and the relevant literature was reviewed to gain insight into LCS. The case raises new questions on the oncogenic character of EBV.

## Introduction

Langerhans cell sarcoma (LCS) is an extremely rare, malignant neoplasm that originates from Langerhans cells (LCs). Fewer than 70 cases have been reported in the English-language literature. LCS typically involves multiple organs, including the skin, lymph nodes, lungs, bone, bone marrow, liver, spleen, and soft tissues. Several etiological factors for LCS have been proposed, including immunosuppression, virus infection, and prior hematological disease (1). We report a rare case of LCS with Epstein–Barr virus (EBV) infection; bilateral cervical giant cysts were the initial manifestation. To our knowledge, this is the first report of LCS with EBV infection. The case information was complete, and the relevant literature was reviewed to gain insight into LCS. The case raises new questions on the oncogenic character of EBV.

## Case Presentation

**Figure 1:**
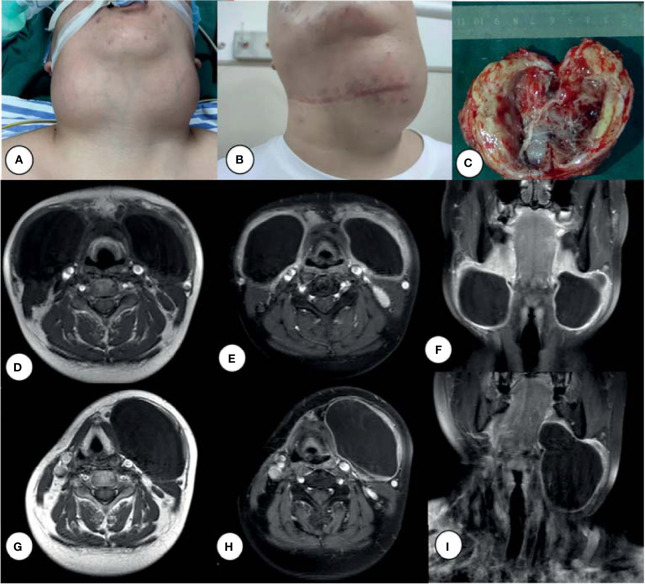
Bilateral cervical giant cysts **(A)**, left cyst enlarged after chemotherapy **(B)**, and surgical excision of the right neck mass **(C)**. MRI of a tumor before surgery **(D–F)** and after **(G–I)** chemotherapy: T1-weighted images **(D, G)**, Ga-enhanced T1-weighted images **(E, F, H, I)**.

A 24-year-old male patient presented with a 6-month history of bilateral neck masses and a sensation of distension when swallowing, and complained that the mass had rapidly enlarged recently. The patient had a medical history that included surgery for fixation of a fracture of the right leg 16 years ago and pulmonary bullae resection 3 years ago. The patient also had a history of smoking (one pack of cigarettes per day for 7 years). Physical examination revealed two tender, fixed, and painless masses with a clear boundary (a ~7- × 6-cm mass in the left submandibular area and an ~8- × 7-cm mass in the right submandibular area; **Figure 1A**). Magnetic resonance imaging (MRI) (**Figures 1D, E**) revealed bilateral cervical cystic lesions and multiple enlarged bilateral cervical lymph nodes.

The patient underwent surgery for excision of a neck mass on April 23, 2020. Grossly, the 8- × 7-cm, pale-yellow mass was identified in the right neck and excised completely along its border up to the skull base. The mass was cystic and filled with thick, dirty yellow fluid, with a ~1-cm-thick cystic wall (**Figure 1C**). Based on the pathological results of proliferative lesions in lymphohematopoietic tissue, the left mass was treated after routine pathological diagnosis. The final pathology report supported a diagnosis of LCS. Following surgery, the patient received two courses of chemotherapy (cyclophosphamide, doxorubicin, vincristine, and prednisone; CHOP). The patient underwent a second surgery on August 1, 2021 because the left lateral cervical mass (**Figure 1B**) was not significantly reduced and pain was experienced when swallowing.

### Pathological Findings

**Figure 2:**
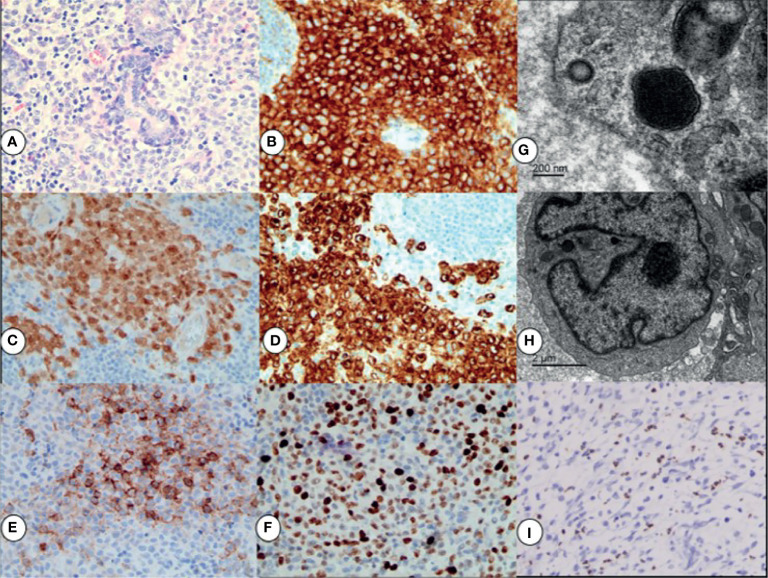
By H&E staining results: the neoplastic cells exhibited cytological atypia, hyperchromatic nuclei, prominent nucleoli, and a high mitotic rate **(A)**. Immunohistochemical staining for CD1a + **(B)**, S-100 + **(C)**, Langerin+ **(D)**, and CD56+ **(E)**; the Ki67 proliferation index was ∼60% **(F)**. All magnifications ×40. Electron micrograph showing a large kidney‐shaped nucleus **(G)** and typical Birbeck granules **(H)** in neoplastic cells. EBER *in situ* hybridization indicating positive signals in the nuclei of background lymphocytes **(I)**, ×20.

The neoplastic cells exhibited cytological atypia, hyperchromatic nuclei, and prominent nucleoli, and nuclear grooving was observed in some of them (**Figure 2A**). Immunohistochemical studies revealed that the malignant tumor cells were positive for CD1a (**Figure 2B**), S-100 protein (**Figure 2C**), and Langerin (**Figure 2D**). There was variable expression of CD56 (**Figure 2E**), cyclin D1, CD4, CD68, and CD163. The proportion of p53 was ~3%. Mitoses were frequently identified, and the Ki-67 proliferative index (**Figure 2F**) was ~60%. Electron microscopy demonstrated the presence of a large kidney‐shaped nucleus (**Figure 2G**) and typical Birbeck granules (**Figure 2H**), with unique striated cytoplasmic organelles characteristic of neoplastic cells.

Pathological findings of the second surgical specimen after chemotherapy showed that CD56, CD68, and CD163 expression became negative in neoplastic cells. Chromogen *in situ* hybridization for the Epstein–Barr encoding region (EBER) of background lymphocytes (**Figure 2I**) was positive. Molecular testing showed that the tumor was negative for BRAF V600E mutations.

### Laboratory Findings

**Figure 3:**
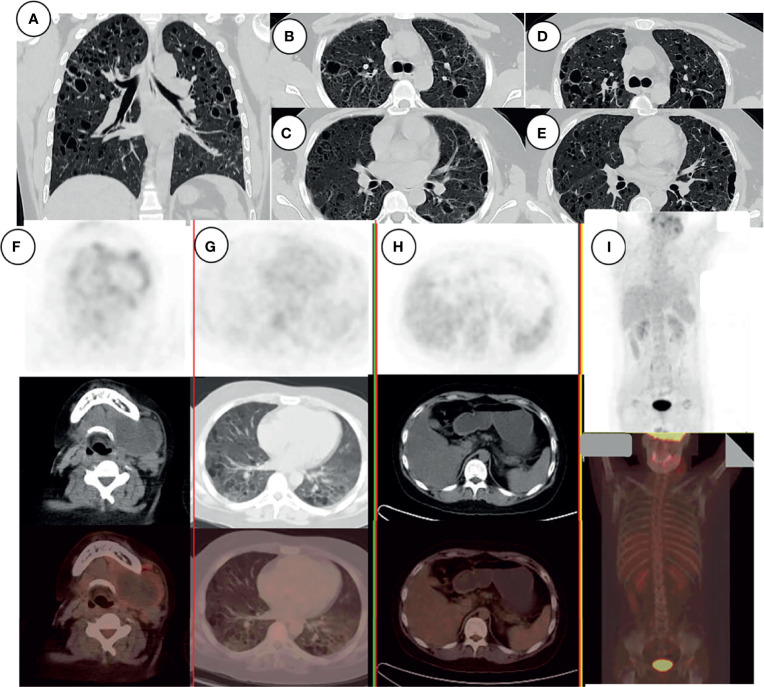
HRCT of the chest, showing numerous variably sized pulmonary cysts that were confluent in some places **(A–C)**; the cysts became larger and thin-walled after chemotherapy **(D, E)**. PET-CT examination of the patient’s general condition. Signal from the left neck mass and multiple small lymph nodes **(F)**, pulmonary cystic lesions **(G)**, and liver **(H)**. There was no signal from bone **(I)**.

The laboratory findings revealed an EBV immunoglobulin (Ig) G of 6.4, human cytomegalovirus (HCMV) IgG of 5.6, and EBV-DNA of 1.05 × 10^3^ copies/ml. The CD3/4/8/16/19/45/56 lymphocyte count was 559 cells/μl (range, 800–4,000 cells/μl), the T-cell count was 276 cells/μl (range, 797–2,370 cells/μl), the helper T-cell count was 138 cells/μl (range, 432–1,341 cells/μl), the killer T-cell count was 125 cells/μl (range, 238–1,075 cells/μl), the natural killer (NK) (CD16+ and CD56+) cell count was 160 cells/μl (range, 127–987 cells/μl), and the B-cell count was 115 cells/μl (range, 86–594 cells/μl). After chemotherapy, the CD3/4/8/16/19/45/56 lymphocyte count was 618 cells/μl, the T-cell count was 278 cells/μl, the helper T-cell count was 98 cells/μl, the killer T-cell count was 155 cells/μl, the NK (CD16+, CD56+) cell count was 305 cells/μl, and the B-cell count was 29 cells/μl. EBV and HCMV capsid antigen IgG, but not IgM, was positive, indicating historic rather than recent EBV and HCMV infection. The EBV DNA load was 1,050 copies/ml. Lymphatic subgroup analysis showed that the patient was in an immunosuppressed state, with reduced T cells and a low CD4/CD8 ratio. After chemotherapy, the CD4/CD8 ratio and B-cell count were lower. High-resolution computed tomography (HRCT) of the chest before chemotherapy (**Figures 3A–C**) showed numerous variably sized pulmonary cysts that were confluent in some places, and HRCT of the chest after chemotherapy (**Figures 3D, E**) showed that the pulmonary cysts were enlarged and thin-walled. MRI of the neck after chemotherapy (**Figures 1G–I**) showed that the cyst had again increased in size and the cystic wall was thinner than before (**Figures 1D–F**). The patient’s general condition was assessed by positron emission tomography-computed tomography (PET-CT) (**Figures 3F–I**). A 90 × 85-mm mass was detected in the left neck with a maximal standardized uptake value (SUV_max_) of ~5.7 (**Figure 3F**); the multiple small lymph nodes located close to the mass had an SUV_max_ of ~5.5. The SUV_max_ of pulmonary cystic lesions was ~1.6 (**Figure 3G**). Fluorine-18 fluorodeoxyglucose (FDG) uptake in the LCS lesions was lower than in prior reports (cases 19, 24, and 25 in **Table 1**). The decreased FDG uptake may be related to chemotherapy.

**Table 1:** Included studies (n = 51).

Case	Author, Date	Sex/age	Site	Histological examination; EM (Birkbeck granules)	Genetic analysis	Diagnostic techniques	Therapy	Out-come	DSS/DFS (months)	Relevant supplementary
1	Wood et al. (2) July	M/71	Skin, LN, lung, liver, spleen, kidney, bladder, myocardium	CD1a+/Ia+/HMC (9–17/mm2); EM: (+)		Sigmoidoscopy, Barium enema, X-ray, BMB,	C (MET/VCR/PDN/TAM)	DOD	2/0	Classified as malignant histiocytosis X
2	Delabie et al. (3) Mar	F/23	Skin, LN, liver, lung	S-100+/Vimentin+/CD68+/α-chymotrypsin+/CD11b+/CD 14+/HLA-DR+/EMA+/acid phosphatase+/non-specific esterase+; EM: (+)	TCR (-), IGH: (-)	X-ray, CT, BMB	S, C (nitrosourea/DPP/VP-16/ADM/MOPP)	DOD	NA/NA	EBV(-), CMV(-) Described as malignant LCT
3	Tani et al. (4) Apr	F/49	Skin, LN, lung, liver, kidney, BM,	CD1+/CD2+/CD3+/CD4+/CD8+/CD11b+/CD21+/HLA-DR+/HMC (16 ~64/mm^2^); EM: (+)		CT	S, C (CTX/PDN/VCR/BLM/IFO/VDS/hydroxyl daunomycin/VP-16)	DOD	43/24	Designated as malignant LCT
4	Lauritzen et al. (5) Jul	M/38	Skin, LN, lung	CD1a+/S100+/CD4+/CD68+/CD11b+/CD11c+/CD13+/CD14+/CDw32+/PNA+/lysozyme+; EM: (+)			C (VCR/proc/must)	AWD	12/0	
5	Itoh et al. (6) Aug	F/74	Skin, LN, lung	CD1a+/S100+/MIB‐1/ki-67(~20%); EM: (+)		X-ray, CT	S, R, C (VLB/VP-16/MP/prednisolone/MST-16)	DOD	14/5	The first experiment of MIB‐1 index in LCH
6	Misery et al. (7) Sep	F/35	Skin	CD1a+/S100+/vimentin+/CD68+/HAM56+/lysozyme+/HMC (20 ~ 70/mm2)/ Ki-67(15%; M: (+)		Ultrasound, CT, MRI, BMB	S	ACR	24/24	Classified as MLC
7	Kawase et al. (8) May	M/59	Skin, LN, lung, BM	CD1a+/S100+/Langerin+/CD4+/CD68+/CD56+/MR (10~40/HPF)		BMB	C (CHOP)	DOD	20/0	
8		M/35	Bone, LN, lung, liver			X-ray, CT, MRI, BMB	C (Ara-c/VCR/PDN/VP-16)	DOD	47/0	
9		F/61	LN			CT, BMB	C (CHOP)	DOD	10/0	Subsequent development of AML
10		M/60	Bone			X-ray, MRI, BMB	R	AWD	11/0	
11	Ferringer et al. (9) Feb	M/33	Skin, LN	CD1a+/S-100+/NSE+/CD31+/MR (~50/10HPF)/Ki-67 (22%); EM: (+)			C (ADM/IFO)	ACR	5/5	CD31 positivity was firstly reported
12	Jülg et al. (10) Mar	M/81	Lung, LN	CD1a+/S100+/Vimentin+/CD68+/CD45+/CD4+/Ki-67(70%); EM:(-)		CT	C (CHOP)	DOD	<1/0	Smoker
13	Lee et al. (11) Jun	M/35	Lung	CD1a+/S-100+/Vimentin+/CD68+/MR (30~60/HPF); EM: (-)	Arising from LCH	CT	S	ACR	5/5	Smoker; a history of pulmonary tuberculosis
14	Lian et al. (12) Nov	F/57	Bone(talus), lung	CD1a+/S100+/Vimentin+/CD68/Ki-6(~40%);		X-rays, CT	S, C, R	DOD	9/0	
15	Bohn et al. (13) Feb	M/47	Skin, LN	CD1a+/S100+/CD207+/vimentin+/CD68+/p53+/MR:(~50/10HPF)/Ki67(60~90%)		CT	S, C (CDA/CTX/VCR/DOX/PDN)	AWD	12/12	
16	Diaz-Sarri et al. (14) Aug	M/58	Skin, LN	CD1a+/S100+/vimentin+/MR (80%); EM: (+)		X-ray, ultrasound	S	ACR	NA/NA	Immunosuppression after LT (Cs A/Allopurinol)
17	Uchida et al. (15) Jan	M/72	Skin	CD1a+/S100+/CD68+/MR (38/10HPF)/ Ki67(53.3%)		MRI, PET	C (MAID), then surgery	ACR	18/18	
18	Sumida et al. (16) 2008 Mar	M/57	LN, tonsil, spleen, BM	CD1a+/S100+/Langerin+/CD4+/CD68+/CD123+/MR (10~20 10HPF); EM: (-)	GR for the TCR or IGH was not identified	CT	C (CTX/VCR/THP/prednisolone/CTX/Ara-c/VP-16)	DOD	7/0	Subsequent development of AML
19	Yoshimi et al. (17) 2008 Jun	F/53	Skin, LN, lung, liver, spleen, stomach, kidney, BM	CD1a+/S100+/Vimentin+/CD68+/MR (80/10HPF)	(EBER–ISH)-	CT, PET	C (CHOP/VP-16/CBR/Arac/prednisolone)	DOD	3/0	Immunosuppression after LT
20	Langfort et al. (18) May	M/47	Lung, LN	CD1a+/S100+/LCA+/CD68+/MR (35/10HPF)/Ki-67(70%; EM: (+)		CT, Gastroscopy	C (PDN/CTX), S	AWD	3/0	Smoker
21	Zhao et al. (19) Aug	F/74	Gallbladder, LN(peritoneal)	CD1a+/S100+/Langerin+/vimentin+/CD4+/P53+/MR(50/10HPF)/Ki-67(70%; EM: (-)		Ultrasound, CT, MRI	S	ACR	8/8	
22	Ratei et al. (20) 2010 Sep	M/21	LN, ileum	CD1a+/S100+/Vimentin+/CD68+/CD45+/HLA-DR+/Ki-67 (50%);	Identical clonal IGH	CT, Colonoscopy,	C (VLB/prednisolone/Arac/MIT), BMT	ACR	36/23	Preceding B-ALL
23	Nakayama et al. (21) Dec,	M/62	LN (neck)	CD1a+/S100+/Langerin+/Fascin +/CD68+/CD163+; EM: (-)		CT, PET	R	ACR	45/45	
24	Musliman et al. (22) Jan	F/69	Pyriform sinus, LN	CD1a+/S-100+;	Identical karyotypes and identical clonal IGH	PET-CT,	C (GEM/DTX)	DOD	1/0	Preceding HCL
25	Yang et al. (23) Jan	M/52	Lung, LN, bone(rib)	CD1a+/S100+/CD68+/MR(>25/10HPF)		X-ray, CT, PET	C (CHOP)	DOD	3/0	Smoker
26	Furmanczk et al. (24) Jun	M/76	Skin, soft tissue, spleen	S100+/CD1a+/langerin+	Identical IGH	MRI, BMB	S, R	DOD	13/5	Preceding HCL
27	Wang et al. (25) Aug	M/41	Skin, LN, lung, liver,	CD1a+/S100+/CD207+/vimentin+/CD68+/Lysozyme/MR (30/10HPF)/Ki-67:(70%-90%)		X-ray,	S, R, C (COP/CHOP)	DOD	<12/2	
28	Xu et al. (26) Sep	M/86	LN, lung, spleen	CD1a+/S100+/langerin+/CD30+/CD4+/p53+/Ki-67 (50%)		CT, FCM, BMB	R	DOD	1/0	CD3 positivity was first reported;
29	Shimizu et al. (27) Nov	F/67	LN	MIB1:(30%)		CT, PET	C (ADM/IFO/MESNA), R	ACR	48/48	
30	Wang et al. (28) Nov	F/77	LN, nasopharynx, lung, spleen	CD1a+/S100+/Langerin+/vimentin+/CD68+/CD163+/Ki-67 (60%); EM:(+)	EBER (-)	CT	Nil	DOD	<1/0	
31	Li et al. (29) Feb	M/48	Skin,	CD1a+/S100+/langerin+/CD68+/Ki-67 (~80%)		PET, BMB	S, C (CHOP)	ACR	12/12	
32	Au et al. (30) Mar	M/21	Skin, LN	CD1a+/S100+/langerin+/MR (frequent)		PET	S, R	ACR	Un/Un	
33	Sagransky et al. (31) 2013 Apr	M/54	Skin	CD1a+/S100+/CD4+/CD31+/CD34+/CD68+/CD83+/MPO	AML revealing a 11:19(+)	BMB	C(DAC/DNR/Ara-c/Ara-c), BMT	ACR	60/60	Preceding AML Trans-differentiation not proven genetically
34		F/63	Skin	CD1a+/S-100+/CD4+/langerin+; EM (+)		BMB	C	DOD	3/0	Preceding unclassifiable MD/MP evolving into AML
35		M/61	Skin	CD1a+/S100+/CD4+/langerin+; EM (+)			S	ACR	Un/Un	
36		M/88	Skin, LN	CD1a+/S100+; EM (-)			S, C	DOD	3/0	
37	Chung et al. (32) 2013, May	F/11 m	LN, lung, liver, spleen, bone	CD1a+/S-100+		CT, MRI, BMB	C (VP-16/DXM, IFO/CBR/VP-16 for recurrent LCS, BMT	ACR	18/16	
38		F/17mon	LN, skin, liver, bone, BM,	CD1a+/S-100+/CD68+		Ultrasound, CT, MRI, BMB	C (VP-16/DXM),; Recurrence: C (CDA/Ara-c), BMT	AWD	24/15	
39	Chen et al. (33) 2013 Jun	F/68	LN	S100+/CD1a+/langerin+/MIB-1(~ 40%)	BRAF V600 (+) Identical 6q23(+)		C (DXM/ADM/Ara-c/CBR)	DOD	Un/Un	Preceding CLL/SLL
40	West et al. (34) 2013 Jul	M/60	LN	CD1a+/S-100+/langerin+/PAX5+	BRAF V600E (-) Identical IGH and IGK GR in LCS		Nil	DOD	3/0	Preceding FL
41	Valentin-Nogueras et al. (35) 2013 July	M/71	Skin, LN, lung	CD1a+/S-100+/MF(Frequent); EM: (+)		X-ray, CT, BMB	S, R, C (CTX/VCR/PDN)	DOD	<24/6	Preceding MDS (lenalidomide 10 mg daily)
42	Keklik et al. (36) Nov	M/39	Nasopharynx, LN	CD1a+/S-100+/CD45+/MR:(~20/10HPF)/ Ki-67(~50%)		PET, BMB	C (2-CDA/ESHAP)	DOD	3/0	Smoker
43	Lee et al. (37) Feb	F/45	Skin, LN, lung	CD1a+/S‐100+/CD68+/CD45+/MR: (>20/10HPF)		CT, PET	C (CHOP), R	AWD	16/0	
44 [12]	Zwerdling et al. (38) 2014. Aug	F/7	Bone (T5-T6)	CD1a+/S-100+/CD68+/vimentin+/CD43+/INI-1/+PGP9.4/MR:(20/10HPF)/ Ki-67:(10%-30%)	BRAF V600E (+)	PET, BMB	S, C (CHOP), R	ACR	17/17	
45	Chang et al. (39) 2014 Aug	F/70	LN	CD1a+/S100+/langerin+/CD68+/Ki-67 (60%)	BRAF V600 (-) CML revealing a BCR-ABL1 fusion		S (LN excision), C (CHOP)	ACR	36/36	Preceding CML (imatinib mesylate); Trans-differentiation not proven genetically.
46	Liu et al. (40) Jun	M/62	Bone (scapula), LN, lung, liver,	CD1a+/S-100+/CD68+/CD163+/CD14+/Fascin+/HLA-DR+/lysozyme+		CT	Nil(refuse)	DOD	<1/0	
47	Zhang et al. (41) Nov	M/75	Soft tissue (knee), LN, liver, omentum	CD1a+/S-100+/CD68+/vimentin+/Ki-67(70%)		MRI, PET	S, R, C (CTX/EPI/HCL/VDS/PDN)	DOD	14/0	A history of CRC
48	Zhang et al. (42) Jun	M/9m	Colonic mucosa, LN,	CD1a+/S-100+/CD207+/Ki67(70%)		Colonoscopy, CT, BMB	NA	NA	NA	The first reported case involvement of the digestive tract in infantile LCS.
49	Yi et al. (43) May	M/41	LN	CD1a+/S-100+/CD207+/CD4+/CD163+/CD68+/vimentin/Ki-67: (65%); EM (+)	Arising from LCH	CT, PET	C (E-CHOP): S	AWD	NA	
50	Tillit et al. (44) May	F/73	Skin (vulva)	CD1a+/S-100+/CD4+/LCA+/CD68+/Ki-67 (85%)	EBER (-); BRAF (-)	CT	S, R	ACR	33/33	Smoker
51	Present case	M/24	LN, lung	CD1a+/S-100+/CD207+/CD56+/Cyclin D1+/CD4+/CD68+/CD163+/p53+/Ki67(60%); EM (+)	EBER (+); BRAF 600E (-)	CT, MRI, PET	S, C (CHOP)	ACR	12/12	Smoker

EBER, Epstein–Barr encoding region; EBER–ISH, Epstein–Barr virus-encoded small RNA 1 in situ hybridization; GR, gene rearrangement; TCR, T-cell receptor; IGH, immunoglobulin heavy chain; LCH, Langerhans cell histiocytosis; LCS, Langerhans cell sarcoma; LCT, Langerhans cell tumor; MLC, malignant Langerhans cell; FL, follicular lymphoma; HCL, hairy cell leukemia; AML, Acute myeloid leukemia; PTC, Papillary thyroid carcinoma; MDS, myelodysplastic syndrome; MD/MP, myelodysplastic/myeloproliferative neoplasm CRC, colorectal cancer; LT, liver transplant; CT, computed tomography; MRI, Magnetic resonance imaging; (FDG–) PET, (fluorine-18 fluorodeoxyglucose) positron emission tomography; BMB, Bone marrow biopsy; S, surgery; R, radiotherapy; C, chemotherapy; Ara-c, cytosine arabinoside; 2-CDA, 2-chlorodeoxyadenosine; IFO, ifosfamide; VDS, vindesine; CTX, cyclophosphamide; MAID, mesna, doxorubicin, ifosfamide, dacarbazine; COP, cyclophosphamide, oncovin and prednisone; CHOP, cyclophosphamide, doxorubicin, vincristine, and prednisone; ESHAP, etoposide, carboplatin, cytarabine, methylprednisolone; E-CHOP, etoposide, cyclophosphamide, vindesine, dexamethasone; BMT, bone marrow transplant; ACR, alive in complete remission; AWD, alive with disease; DOD, died of disease; DSS, disease specific survival; DFS, disease free survival.

## Discussion

LCS is an extremely rare neoplastic proliferation of LCs with overtly malignant cytological features and unusually aggressive behavior. In the classification of tumors of the hematopoietic and lymphoid systems of the World Health Organization (WHO) (WHO-2016) (1), LCS is defined as a neoplastic disorder of LC with apparent malignant cytological features, possibly including both LCS progressed from Langerhans cell histiocytosis (LCH) and *de novo* LCS. LCS is distinguished from LCH, which is also involves the neoplastic proliferation of cells, in terms of its immunophenotypic and electron microscopic features of LC, cytologic atypia, and clinical aggressiveness (45). However, it can be difficult to classify a lesion as LCH or LCS. LCS displays typical features of malignant tumors and usually involves multiple organs, including the skin, lymph nodes, lungs, liver, spleen, kidneys, bone, bone marrow, and other soft tissues.

We conducted a systematic literature review on LCS from 1984 to December 2020 (keywords: Langerhans cell sarcoma), focusing on studies describing the etiology and pathology of LCS. The available reports are summarized, together with the present case, in **Table 1**. These cases (Cases 14, 17, 19, 22, 24, 25, 29, 33, 37, 38, 42, 43, 44, 46, 47) having no CD207 (langerin) or electron microscopic features of LCs, might not meet all the current criteria in WHO-2016 for LCS sarcoma diagnosis. LCS can occur at any age, with patients ranging from 9 months to 88 years old. As shown in **Table 2**, the male-to-female ratio was 1.68. In 7.9% of cases (n = 4), the primary site at diagnosis of LCS was the lungs; all four of those patients were smokers. Interestingly, pulmonary LCH is almost always associated with smoking (46). Liu (47) provided mechanistic insight into the role of tobacco smoke in the development of pulmonary Langerhans cell histiocytosis (PLCH) using a smoking mouse model. However, only a few of the reported cases of primary lung LCS considered the smoking history. The most common primary site at diagnosis was the skin among the cases reviewed herein (24 cases, 47.1%), followed by the lymph nodes (13 cases, 25.5%), bone (5 cases, 9.8%), and lung (4 cases, 7.9%; 1 case each in the gallbladder, pyriform sinus, nasopharynx, colonic mucosa, and soft tissue). At diagnosis, 25.5% of cases had local disease, 23.5% had locoregional disease, and 51.0% disseminated disease. The most common sites were the lymph nodes (40 cases, 78.4%) and skin (26 cases, 51.0%), followed by the lung (20 cases, 29.2%) and the other organs listed in **Table 2**.

**Table 2:** Patient characteristics.

Variables	Number of Patients
Gender	
Male	32
Female	19
Primary site	
Skin	24
Lymph node	13
Bone	5
Lung	4
Gallbladder/pyriform sinus/nasopharynx/soft tissue/colonic mucosa	1
Site	
Lymph node	40
Skin	26
Lung	20
Liver	9
Bone	8
Spleen	7
Bone marrow	5
Kidney	3
Nasopharynx/soft tissue	2
Bladder/myocardium/tonsil/stomach/ileum/omentum/gallbladder/pyriform sinus/colonic mucosa	1
Associated factors	
Smoking	7
Not reported	44
Arising from LCH	2 (case 13, 49)
EBV	
Positive	1 [case 51 (present)]
Negative	4 (case 2, 19, 31, 50)
Not reported	46
BRAF	
Positive	2 (case 39, 44)
Negative	4 (case 40,45,50,51)
Not reported	45
Long-term immunosuppressive	2 (case 16, 19)
Prior hematological disease	9 (case 22, 24, 26, 33, 34 39, 40, 41,45)
Leukemic transformation	2 (case 9, 18)
Trans-differentiation proven genetically	5 (case 22, 24, 26, 39, 40)

The rarity of LCS hampers investigation of its pathogenesis. Several etiological factors have been proposed, including immunosuppression, prior hematological disease, and virus infection. Immunosuppression has been linked to increased rates of malignancy (2.7- to 13.7-fold increase post-transplant) with the risk increasing with the intensity and duration of the immunosuppression. Our review revealed two LCS cases (17, 48) occurring against a background of immunosuppression for previous liver transplants (cases 16 and 19). Furthermore, Rate’s LCS (20) case was controlled only by stopping the immunosuppression (case 22). Long-term immunosuppressive treatment after organ transplantation may promote the development of LCS.

Some LCS cases have been linked to prior hematological disease. LCS may be preceded by acute B-lymphoblastic leukemia (B-ALL) (20) (case 22), hairy cell leukemia (HCL) (49, 50) (cases 24 and 26), acute myeloid leukemia (AML) (31) (case 33), unclassifiable myelodysplastic/myeloproliferative neoplasm (MD/MP) evolving into AML (38) (case 44), chronic lymphocytic leukemia/small lymphocytic lymphoma (CLL/SLL) (33) (case 39), follicular lymphoma (FL) (34) (case 40), myelodysplastic syndrome (MDS) (35) (case 41), and chronic myelogenous leukemia (CML) (39) (case 45). Two cases showed leukemic transformation (8, 16) (cases 9 and 18). In summary, LCS can occur in association with other hematological disorders (20, 33, 34, 49, 50) (cases 22, 24, 26, 39, and 40) to which it is clonally related. These cases carry the same T-cell receptor (TCR) or Ig heavy chain (IGH) gene rearrangements and chromosomal aberrations as the associated lymphoid neoplasms, suggesting a process of transdifferentiation [WHO-2016 (1)]. LCS can exhibit acute leukemic transformation, and a wide variety of clonal malignancies can transdifferentiate into LCS.

Viral infections are associated with approximately 12% of all cancers worldwide. LCs are present beyond the middle of the spinous epidermal layer and function as sentinel or antigen-presenting cells that can capture invading viruses (51). The interaction between LCs and viruses results in highly variable responses. The inflammatory nature of LCH lesions raises the possibility that infection and immune dysregulation may be the mechanisms of pathogenesis (52). Several viruses have been studied as potential etiological factors of LCH, including EBV (53), human herpesvirus 6 (HHV-6) (54), cytomegalovirus (CMV) (55), herpes simplex virus (HSV) (56), and Merkel-cell polyoma virus (MCV or MCPyV) (57). Murakami reported that MCV-related molecules are present in more than half of LCH cases, and in some dermatopathy lymphadenopathy cases (58), and that three LCS cases were positive for viral DNA sequences (59). It was postulated that a high MCV load in LCS lesions is an important oncogenic factor in LCS cells. EBV is the etiological agent in several malignancies and may play a role in the pathogenesis of LCH (60, 61). The main reservoir of latent EBV infection *in vivo* is the B-lymphocyte population. EBV latently infects a unique subset of blood-borne mononuclear cells that are direct precursors of LCs derived from B lymphocytes and could be reactivated and replicated in LCs (62, 63). Daniel revealed that Hodgkin lymphoma (HL) with excess Langerhans cell shows greater LMP1/EBV expression, which may increase cytokine production by activating nuclear factor kappa B (NF-κB), and thus explain the abundance of LCs (64). Therefore, evidence of EBV infection in LCS would be interesting, and the association of EBV infection with LCS should be investigated.

In our case, the patient had an EBV-DNA level of 1.05 × 10^3^, and chromogen *in situ* hybridization for EBER of background lymphocytes was positive. This is the first reported LCS case positive for EBV markers. Lymphatic subgroup analysis (CD3/4/8/16/19/45/56) showed that the patient was in an immunosuppressive state with reduced T cells and a low CD4/CD8 ratio. Allograft recipients given T-cell-suppressive drugs to prevent graft rejection and HIV-infected individuals who progress to profound T-lymphopenia and late-stage acquired immunodeficiency syndrome (AIDS) provide the clearest evidence of a key role for T cells in the control of EBV-induced disease (65). Profound T-cell depletion of the allograft represents a major risk factor for EBV-induced post-transplantation lymphoproliferative disorder (EBV-PTLD), which is a life-threatening complication of allogeneic hematopoietic cell transplantation (66). In the absence of T-cell control, the lymphoproliferative disease seen in late-stage AIDS is the equivalent of classical PTLD, which is characterized by the growth of EBV-transformed lymphoblastoid cell line (LCL)-like cells, often in the central nervous system (65). EBV-associated smooth muscle tumors (SMTs) are rare malignancies that occur exclusively in immunocompromised patients, typically due to posttransplant immunosuppression or HIV infection (67). Moreover, immunosuppressants, including methotrexate (MTX) and tacrolimus (TAC), are widely used to treat patients with rheumatoid arthritis (RA), and their adverse effects have been known to cause other iatrogenic immunodeficiency-associated lymphoproliferative disorders (OIIA-LPDs). Seiji reported that the tumor cells were positive for EBV in 8 (17%) of 48 patients; background cells were positive in 32 (82%) of 39 patients with available data in the literature review of MTX-associated T-LPDs (MTX T-LPDs) (68). The presence of EBV reflects a profound immunodeficiency and may drive the development or a rapid progression of the tumor.

We postulated that our patient likely developed LCS due to EBV infection under conditions of congenital or acquired immunosuppression *via* a mechanism similar to EBV-PTLD, EBV-positive SMTs (EBV + SMTs), or MTX T-LPDs. The case raises new questions regarding the oncogenic nature of EBV.

The pathological results were consistent with LCS. Immunohistochemistry was performed on samples obtained before and after chemotherapy. The expression of CD56, CD68, and CD163 became negative in the neoplastic cells after chemotherapy, possibly attributable to the effectiveness of chemotherapy. Some experts have recommended using CD56 as a marker for differential diagnosis of LCS and LCH. Kawase found that tumor cells in all four cases of LCS in their study were positive for CD56 (8), whereas the tumor cells in all eight cases of LCH were negative. Our findings indicated that CD56 may be a clinically relevant predictor of an intractable course of LCS. The present case was negative for the BRAF V600E mutation, which involves a molecular change underlying the pathogenesis of many malignancies. Almost half of all cases of LCH reportedly harbor the BRAF V600E mutation (69), while only two of the cases of LCS reviewed herein (33, 38) (cases 39 and 44) had the BRAF V600E mutation. Given the poor outcomes of LCS, we suggest that immunohistochemical testing for the BRAF mutation should be performed. Vemurafenib, a BRAF inhibitor, may have therapeutic potential, especially in older individuals in whom combined therapy is expected to be poorly tolerated (38) (case 44).

Because of its rarity, the optimal treatment strategy for LCS has not been established, and treatment depends on the affected site and scope (**Table 3**). For localized nodular disease, one patient (case 40) with a history of FL received no therapy due to severe disease progression, 84.6% (11/13) achieved complete remission with monotherapy (only surgery was used in cases 6, 13, and 35; only radiotherapy was performed in case 23) or multimodal therapy, and 7.7% (1/13) receiving only radiotherapy were alive with disease at the last follow-up. For locoregional disease, 33.3% (4/12) achieved complete remission with monotherapy (only surgery was used in cases 16 and 21; only chemotherapy was performed in case 11) and multimodal therapy (case 32 received surgery with adjuvant chemotherapy), 25% (3/12) were alive with disease at the last follow-up (cases 15 and 20 received surgery with adjuvant chemotherapy, case 49 received chemoradiotherapy), and 41.7% (5/12) died from their disease (only chemotherapy was performed in cases 24, 25, 34, and 42; only surgery was performed in case 36). Among the patients with disseminated disease, 12% (3/25) achieved complete remission [cases 22 and 37 received a bone marrow transplant (BMT) after chemotherapy and case 51 received surgery with adjuvant chemotherapy], 12% (3/25) were alive with disease at the last follow-up (only chemotherapy was performed in case 11; case 43 received surgery with adjuvant chemotherapy; case 13 received a BMT after chemotherapy), and 76% (19/25) died from their disease. For local or locoregional disease restricted to the skin and lymph nodes, there were good outcomes with all treatment modalities. Of the 25 cases of disseminated LCS, only 3 achieved complete remission at the last follow-up. One patient with disease restricted to the lung and cervical lymph nodes underwent surgery with adjuvant chemotherapy, achieving complete remission. The remaining two patients were treated with BMT; one developed recurrence at 15 months but was alive at 24 months, and the other was cured after 18 months (case 37). Despite receiving conventional combination chemotherapy, surgery, and radiotherapy, 76% (19/25) of disseminated LCS patients showed a poor prognosis and a short survival period because these patients typically have multiple organ involvement and distant metastasis. A patient with disease in more than two organs at diagnosis was reported by Chung (32) (case 37) and achieved complete remission, demonstrating that BMT is the only effective treatment for disseminated LCS.

**Table 3:** Outcomes by individual management strategies.

Modality	Extent at diagnosis	Local (n = 13)			Locoregional (n = 12)			Disseminated (n = 25)		
ALL (n = 50)	Outcome	ACR	AWD	DOD	ACR	AWD	DOD	ACR	AWD	DOD
Surgery (n = 6)		3			2		1			
Primary radiotherapy (n = 4)		1	1							1
Primary chemotherapy (n = 14)					1		4		1	8
Surgery +radiotherapy(n = 4)		1			1					1
Surgery + chemotherapy (n = 8)		3				2		1	1	2
Primary chemoradiotherapy (n = 3)		1				1				
Surgery + chemoradiotherapy (n = 6)		1								5
Bone marrow transplant (n = 4)		1						2	1	
Nil (n = 3)				1						2

ACR, alive in complete remission; AWD, alive with disease; DOD, died of disease.

For LCS with BRAF V600E mutations, vemurafenib, a BRAF V600E mutant inhibitor, has shown efficacy as a targeted, alternative treatment (69). Although the association of EBV and LCS is unclear, patients with high EBV loads may be candidates for antiviral therapy. A reduction in viral load may prevent the development of diseases such as PTLD during primary infection, in addition to other malignant diseases associated with latency (70). Smoking cessation is an important recommendation for smokers with LCS, given that it leads to partial regression in around half of patients with isolated PLCH (46). The present case is particularly uncommon in that the patient developed LCS with EBV infection of the bilateral cervical giant cysts and lung lesions and was treated by a combination of surgery, an anthracycline-containing regimen (ACR) and CHOP chemotherapy, after declining antiviral therapy.

**Figure 4:**
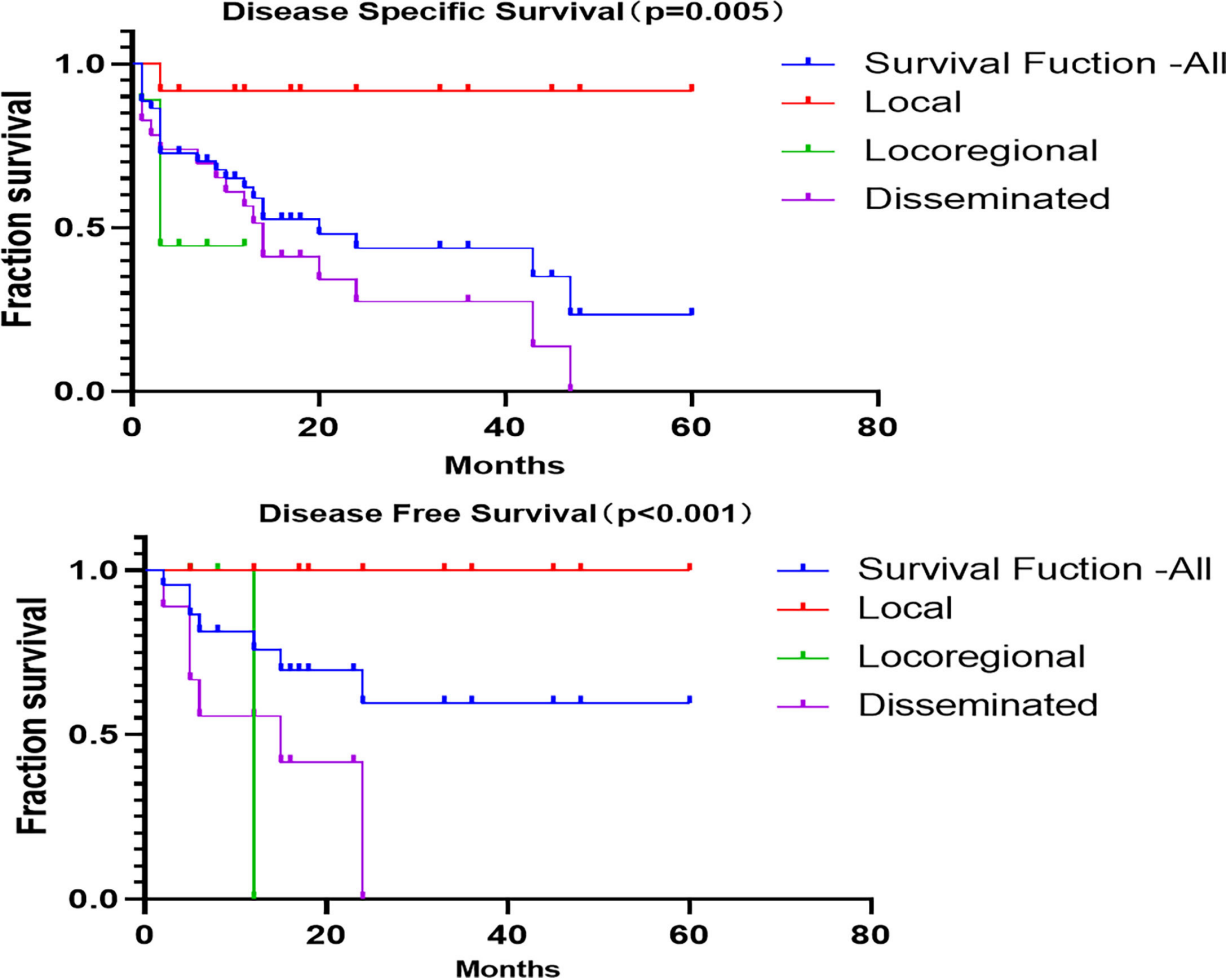
Kaplan–Meier survival curves.

The 1-, 3-, and 5-year disease-specific survival (DSS) for all of the patients with LCS was 54.6%, 15.9%, and 2.3%, respectively. Unfortunately, none of the patients with locoregional disease survived to 3 years, and none with disseminated disease survived to 5 years (**Figure 4**). The 1-, 3-, and 5-year disease-free survival (DFS) for all patients with LCS was 68.2%, 18.2%, and 2.3%, respectively. Unfortunately, none of the patients with locoregional and disseminated disease survived to 3 years. The overall DSS was 28.06 months, with a DFS of 21.22 months. There were significant differences in DSS and DFS among the local, locoregional, and disseminated disease cohorts (p = 0.005 and p < 0.001 respectively; **Table 4**).

**Table 4:** Disease-specific and disease-free survival calculations from Kaplan–Meier survival analysis.

	Disease Specific Survival (Mean)			Disease Free Survival (Mean)		
	Estimate	Std Error	Mantel–Cox	Estimate	Std Error	Mantel–Cox
Overall	28.064	4.136	p = 0.005	21.222	4.354	p < 0.001
Local	55.250	4.548		55.000	4.787	
Locoregional	6.778	1.570		4.000	2.037	
Disseminated	19.497	3.908		5.511	1.920	

In summary, LCS has a poor prognosis and requires pathological diagnosis because of its non-specific clinical manifestations and imaging findings. We reported a rare EBV-positive LCS with bilateral lateral cervical giant cysts as the initial manifestation. The case information was complete, and relevant literature was reviewed to gain insight into LCS. The case raises new questions regarding the oncogenic nature of EBV.

## Data Availability Statement

The original contributions presented in the study are included in the article/supplementary material. Further inquiries can be directed to the corresponding author.

## Ethics Statement

The studies involving human participants were reviewed and approved by the ethics committee of the First Affiliated Hospital, Zhejiang University School of Medicine. The patients/participants provided their written informed consent to participate in this study. Written informed consent was obtained from the individual(s) for the publication of any potentially identifiable images or data included in this article.

## Author Contributions

YG and S-HZ designed and wrote the manuscript. Z-ZC, Y-YB, and L-FS reviewed the references and made the tables. H-TY made the immunohistochemical pictures. All authors contributed to the article and approved the submitted version.

## Funding

The work was supported by the Center of Electron Microscopy of Zhejiang University and State Key Laboratory for Diagnosis and Treatment of Infectious Diseases of Zhejiang University. We obtain the patient’s appropriate consents, permissions, and releases about personal disease details and images for publication.

## Conflict of Interest

The authors declare that the research was conducted in the absence of any commercial or financial relationships that could be construed as a potential conflict of interest.

## Publisher’s Note

All claims expressed in this article are solely those of the authors and do not necessarily represent those of their affiliated organizations, or those of the publisher, the editors and the reviewers. Any product that may be evaluated in this article, or claim that may be made by its manufacturer, is not guaranteed or endorsed by the publisher.
